# From Theory to Practice: A Transtheoretical Treatment and Training Model (4TM)

**DOI:** 10.32872/cpe.12421

**Published:** 2024-04-26

**Authors:** Wolfgang Lutz, Brian Schwartz, Anne-Katharina Deisenhofer, Jana Schaffrath, Steffen T. Eberhardt, Jana Bommer, Antonia Vehlen, Danilo Moggia, Kaitlyn Poster, Birgit Weinmann-Lutz, Julian A. Rubel, Miriam I. Hehlmann

**Affiliations:** 1 Department of Psychology, Trier University, Trier, Germany; 2 Department of Psychology, Osnabrück University, Osnabrück, Germany; Philipps-University of Marburg, Marburg, Germany

**Keywords:** transtheoretical clinical practice and training, psychotherapy outcome research, outcome monitoring, therapist effects, process research, clinical training, precision mental health

## Abstract

**Background:**

In this paper, we present the conceptual background and clinical implications of a research-based transtheoretical treatment and training model (4TM).

**Method:**

The model implements findings from psychotherapy outcome, process, and feedback research into a clinical and training framework that is open to future research.

**Results:**

The framework is based on interventions targeting patient processes on a behavioral, cognitive, emotional, motivational, interpersonal, and systemic/socio-cultural level. The 4TM also includes a data-based decision support and feedback system called the Trier Treatment Navigator (TTN).

**Conclusion:**

We discuss important problems associated with clinical orientations solely based on one school of thought. We then contrast these concerns with a clinical and training framework that embraces ongoing research, serving as a guiding structure for process-based transtheoretical interventions. Such research-based psychological therapy can take both traditional and novel clinical developments as well as findings from psychotherapy research into account and be adaptively disseminated to a variety of patient populations.

## Highlights

- We introduce a research-based transtheoretical treatment and training model (4TM).
- The framework can serve as a guiding structure for process-based transtheoretical interventions.
- It is targeting processes on a behavioral, cognitive, emotional, motivational, interpersonal, and systemic/socio-cultural level.
- It includes a data-informed decision support and feedback system (i.e., Trier Treatment Navigator, TTN).

In this paper, our aim is to introduce the conceptual background and clinical implications of a research-based **t**rans**t**heoretical **t**reatment and **t**raining model (4TM). We discuss important problems associated with clinical theoretical orientations solely based on one treatment approach (or school of thought). These challenges are then met with a clinical and training framework that is open to future research as a guide to process-based transdiagnostic and transtheoretical interventions. At the core of this framework is a data-informed decision system designed to facilitate therapists’ evidence-based clinical decision making throughout the entire treatment process.

Decades of research on treatment effects have provided substantial evidence for psychotherapy as a (cost-)effective intervention for a wide range of psychological disorders (e.g., Barkham & Lambert, 2021). The widespread acceptance and integration of psychotherapy (now often referred to as *psychological therapy* to include the various newer theoretical concepts) into healthcare systems worldwide has led to the establishment of standardized training and certification requirements for therapists in numerous countries (e.g., Lutz, Castonguay, et al., 2021).

In the introduction to the first edition of Bergin and Garfield’s Handbook of Psychotherapy and Behavior Change, Urban and Ford (1971) already noted that the evolution of psychotherapy was not a linear or continuous process. While the field emerged in the late 19th century, its development did not follow a simple linear progression towards ever more refined and effective psychological techniques, strategies, or principles of change. Instead, with the introduction of numerous variations of psychotherapeutic treatments and orientations, progress took several lateral paths. Therefore, it comes as no surprise that even a brief search on Wikipedia using the term “list of psychotherapies” yields 171 variants. However, not all approaches have been empirically studied (Barkham & Lambert, 2021).

Regardless of the presence of an evidence base, a multitude of approaches and therapeutic schools have emerged, each with their own psychopathological and change concepts and corresponding professional organizations. As a consequence, the landscape of accepted approaches and regulations for clinical training and practice has become highly diverse, both within and between countries. Decisions about which treatment approaches are available within a healthcare system or how many sessions per treatment are funded are often based on a mixture of empirical and political considerations (e.g., Lutz, Castonguay, et al., 2021).

Furthermore, for therapists, the different theories seem to constitute a narrative, which gives them a deep sense of meaning and identity and strong feelings of affiliation to a specific therapeutic orientation. Simultaneously, the strong adherence to specific schools of thought within the different approaches has led to rigidity and ongoing disputes among colleagues, often resulting in a narrowed scientific and clinical perspective. It has been common for proponents of a particular therapy school to mistakenly assume that evidence of therapeutic effectiveness automatically validates the underlying theoretical assumptions pertaining to psychopathology and psychological processes of change. However, Rosenzweig (1938) already pointed out this logical fallacy and the patients’ perspective seems to differ. While some patients may find it important to know the theoretical orientation of their therapist, for the majority of patients, their primary concern lies in their improvement through therapy. Patients often recall having engaged in some form of talking therapy but tend not to retain specific details regarding their therapist’s treatment model.

Nevertheless, while this strong therapist identification with a single treatment approach is historically understandable, it is somewhat surprising from a scientific point of view. This is because heterogeneity within treatment schools is enormous, making it challenging to establish a unified set of concepts or mechanisms that are coherent across all variants within a specific treatment orientation. Furthermore, none of the therapeutic orientations have yet established a scientifically grounded and clearly defined causal connection between their theoretically proposed mechanisms of change and treatment outcomes (e.g., Crits-Christoph et al., 2021; Kazantzis et al., 2018).

## Research Guiding Clinical Practice and Training

In the following, six lines of psychotherapy research are described that can form the backbone of a clinical practice and training framework that is open to future developments: 1) comparative outcome research; 2) patient-focused feedback research; 3) research on therapist effects; 4) research on processes and mediators; 5) research on predictors and moderators; 6) research on dissemination and implementation.

Traditionally, outcome-oriented research in psychotherapy has focused on determining the average effectiveness or average comparative effectiveness of specific psychotherapeutic models for particular disorders. This line of research was essential to fostering a widespread acceptance of psychological interventions worldwide. However, this approach has limitations as it solely relies on average or comparative average effects between varyingly well-defined treatment models. Categorizing the change processes in different variations or orientations of psychological therapy can be challenging and is often based on arbitrary boundaries rather than well operationalized theoretical distinctions. Furthermore, the emphasis on specific change processes in certain treatment models and the assumptions regarding their prevalence in a model are often not well studied or understood (e.g., Baldwin & Imel, 2020; Cohen et al., 2023).

Regardless of the long-running controversy surrounding the extent of small or non-existent treatment differences between therapeutic models (e.g., Barkham & Lambert, 2021), for our purpose, two conclusions can be drawn from this line of research: a) Comparative outcome research on treatment procedures and methods does not automatically lead to differentiated clinical knowledge about treatment options for a patient with a particular disorder; b) psychological treatments do not work for all patients and under all circumstances. Negative treatment responses or patients at risk of unfavorable treatment outcomes are usually overlooked in clinical trials and meta-analytic reviews that focus on average effects (Lutz, Schwartz, & Delgadillo, 2022).

Another line of outcome research, patient-focused feedback research, has been an influential research topic in psychotherapy research in the last two decades (e.g., Lutz, Schwartz, & Delgadillo, 2022). Over 50 studies on feedback and routine outcome monitoring (ROM) have been conducted during this period (Barkham et al., 2023). The overall effect of feedback-informed treatments vs. evidence-based treatments without feedback is significant with an effect size of approximately *d* = 0.15 and 8% higher success rates in comparison to treatments not informed by feedback. The application of such a low-cost intervention as continuous measurement and feedback seems to result in improved treatment outcome, reduced dropout, and higher treatment efficiency than standard evidence-based treatments alone. It is important to note that these effects are additional to the effects of well-established evidence-based treatments.

Patients who benefited most from feedback systems were those who, at some point during treatment, indicated a higher risk for treatment failure. The effect can be enhanced when clinical support tools (CSTs) are used to personalize treatment for such “not on track” cases (with an effect size between 0.36 and 0.53 and an average success rate advantage of ~20% to 29%; Barkham et al., 2023). These tools include additional clinical information to adapt treatment specifically to patients at risk for treatment failure by assessing potential problem areas (e.g., motivation, social support, etc.) and then directing therapists to additional interventions for identified risk profiles via a decision tree. New developments include machine learning prediction models and multimedia instruction materials to help therapists provide those interventions, which are the most promising for a particular patient (e.g., Lutz, Deisenhofer, et al., 2022). However, the effects of such systems seem to depend on the extent to which therapists make use of the information provided by feedback systems.

As a summary, this line of research has important implications for clinical practice and training. Feeding psychometric information back to therapists and integrating this procedure into clinical practice via modern technologies seems to have the potential to improve clinical practice, but the effects depend on the quality of its implementation. Therefore, it is important to focus on the use of feedback in clinical training (e.g., Barkham et al., 2023).

Another area of research relevant to transtheoretical interventions is the research on therapist effects, which has shown that therapists’ effectiveness differs systematically independent of their theoretical orientation. The impact of therapist effects on therapy outcomes is estimated to be 5–8% (e.g., Wampold & Owen, 2021), while about 1/8 of therapists have significantly better and 1/9 have significantly worse therapy outcomes than the average therapist. Furthermore, therapist effects are particularly high for more distressed patients. Besides outcomes, therapists also differ regarding therapy duration, dropout rates, and sudden gains (e.g., Lutz, de Jong, et al., 2021).

In addition to therapy outcome, change processes and mediators associated with treatment outcome in psychological therapies have been studied (i.e., “process research”). The first empirically based taxonomies summarizing process–outcome research findings and mediators appeared as early as the 1970s. While it is beyond the scope of this paper to provide a comprehensive summary of the findings and challenges spanning five decades of research, for our purpose, it can be concluded that the outcomes of these investigations vary across different theories and treatment approaches. Further, there is no consensus on the importance of specific core processes or mechanisms of change. Moreover, there are measurement problems and the theoretical constructs have yet to be well validated. In summary, process–outcome research in psychological therapy has not yet been able to demonstrate clear causal relationships between specific mediators, mechanisms, or process variables and treatment outcome (e.g., Cohen et al., 2023; Crits-Christoph et al., 2021).

**Figure 1:**
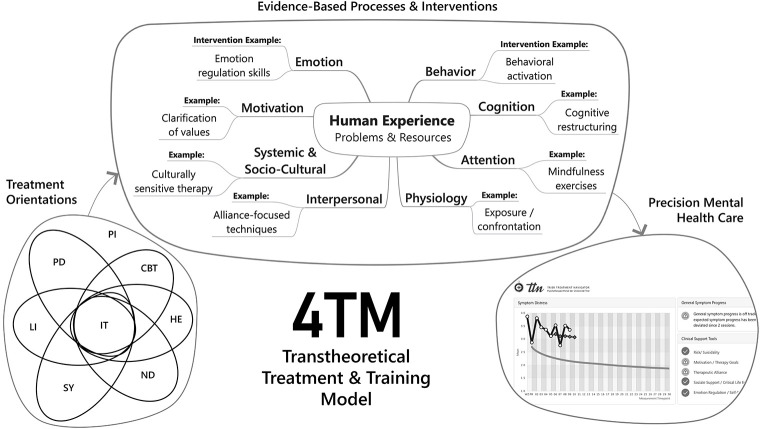
Core Elements of a Transtheoretical Treatment and Training Model (4TM) *Note.* PI = psychological interventions; CBT = cognitive behavioral therapies; PD = psychodynamic therapies; SY = systemic therapies; HE = humanistic-experiential therapies; ND = new developments/third wave (e.g., Acceptance and Commitment Therapy, ACT; Mindfulness-Based Stress Reduction, MBSR; Dialectical Behavior Therapy, DBT; Emotion-Focused Therapy; Mentalization; Positive Psychology); IT = integrative treatment models (e.g., Systematic Treatment Selection, STS; Alliance-Focused Therapy, AFT; Schema Therapy; Cognitive Behavioral Analysis System of Psychotherapy, CBASP); LI = low-intensity treatments (e.g., brief treatments, one session treatments, online therapy). TTN = Trier Treatment Navigator.

Until a clear causal link or network of interconnected strategies and processes is established, all clinical interventions experience some degree of empirical uncertainty. This also includes situations, in which certain interventions might affect multiple processes, and in which some interventions might lack unique contributions, but could be substituted with others that produce a similar effect (see Figure 1). However, numerous investigations have enabled the identification of a wide array of intensively-studied processes/interventions and change principles. While not firmly established in terms of causal connections, these processes and interventions have demonstrated empirical validity (studied using experimental designs, correlational designs, or Granger causality in time series) and maintain clinical relevance in the treatment of a wide range of disorders. The scope of this statement paper does not permit the description of detailed evidence for each of these processes and interventions. However, this line of research can provide general guidance within a clinical and training framework that remains open to future investigations into mechanisms and processes (e.g., Caspar, 2019; Cohen et al., 2023; Crits-Christoph et al., 2021; Eubanks & Goldfried, 2019; Grawe, 2004; Hofmann & Hayes, 2019; Kazantzis et al., 2018; Norcross & Lambert, 2019; Rubel et al., 2017; Wolitzky-Taylor et al., 2023). Over the years, several transtheoretical and integrative frameworks have been developed following this line of research (several examples are given in papers within this special issue).

The treatment strategies and clinical processes described in these models are heterogeneous in their conceptualization and application of research findings and do not stem from a fully empirically-defined network of causally linked elements. However, these concepts are all designed to move beyond the traditional view of categorical diagnosis as the sole basis for treatment selection. Such broader conceptualizations can therefore help to unpack traditional treatment packages and understand clinical practice as a framework of scientifically grounded processes, mechanisms, and strategies, which is open to future research findings.

These empirically studied processes and transtheoretical guidelines can also be related to research on predictors and moderators of change (Barkham & Lambert, 2021). Predictor and moderator models that support the assignment of patients to different treatment options have a long history in psychotherapy research. Over the years, such efforts have become increasingly sophisticated, including new statistical tools based on machine learning algorithms that are closely linked to the development of personalized or precision mental health interventions (Delgadillo & Lutz, in press).

Finally, disseminating psychological treatments constitutes a challenge in many areas of the world, especially in low- and middle-income countries where access to mental health treatments is limited. Transtheoretical concepts can be used to design cost-efficient programs aimed to target specific mental health issues. Various programs have been developed, which include low-intensity, single-session, or internet interventions. These treatment approaches are often rooted in transtheoretical concepts, emphasizing activities that foster engagement (e.g., collaboration, empathy, active listening), as well as addressing various domains of behavior, interpersonal relationships, emotions, and cognitions (e.g., Singla et al., 2017).

It is important to note that these lines of psychotherapy research, which form the foundation of a transtheoretical framework, are not a rigid set of findings and a corresponding treatment approach, but rather a set of concepts that remains open to future research findings and further verifications. Nonetheless, in our opinion, it provides sufficient guidance to structure clinical training and practice effectively.

## What Could Transtheoretical Clinical Practice and Training Look Like?

The open framework introduced above can be built on interventions targeting human experiences that facilitate change processes on a behavioral (e.g., behavioral activation), cognitive (e.g., cognitive restructuring), emotional (e.g., emotion-focused techniques), motivational (e.g., work on life goals), interpersonal (e.g., building a therapeutic relationship), and systemic/socio-cultural level (e.g., strength-based methods, cultural adaptations). Figure 1 illustrates the core elements of such a clinical and training framework that is open to future research, showing the bio-psycho-social network of human experience and the web of associated problems and resources as the targets of evidence-based clinical interventions. Each such intervention might therefore have a broader impact on the entire network (e.g., Fried et al., 2022; Orlinsky & Howard, 1995).

Figure 1 does not include details of evidence-based processes and interventions (see e.g., Lutz & Rief, 2022; Lutz et al., in press), however this concept can guide research-based psychological therapy, while respecting the traditions of successful clinical developments and research within the specific orientations. It includes clinical skills on the micro level, techniques and strategies on the meso level, as well as principles of change on the macro level. The general clinical practice and training framework comprises several tasks and goals of therapy, such as facilitating the acquisition of cognitive, behavioral, and emotional coping skills, strengthening patients’ resources, establishing a therapeutic alliance as a healing context, as well as fostering corrective experiences (on a motivational as well as an interpersonal/cultural/context level) and the acquisition of a more flexible and healthy view of the self and others. A corresponding model of psychological distress posits a transdiagnostic approach to psychopathology. Of course, such a transtheoretical clinical practice and training framework must be continuously updated based on new research findings.

## Example of a Transtheoretical Treatment and Training Model (4TM)

Over the last decade, we have tried to implement such a research-based **t**rans**t**heoretical **t**reatment and **t**raining model (4TM) at the outpatient center at the University of Trier. This 4TM aimed to realize one primary objective: integrating psychotherapy research into clinical training and practice on an ongoing basis. This aim has led to the development of the Trier Treatment Navigator (TTN), a tool that supports and enhances research, training, and practice, fostering synergistic effects between the three (right side of Figure 1). The TTN was successfully evaluated in a recently published prospective randomized-controlled trial (Lutz, Deisenhofer, et al., 2022). It enables continuous monitoring of patient progress via psychometric questionnaires and provides therapists with valuable feedback and clinical decision support tools.

**Figure 2:**
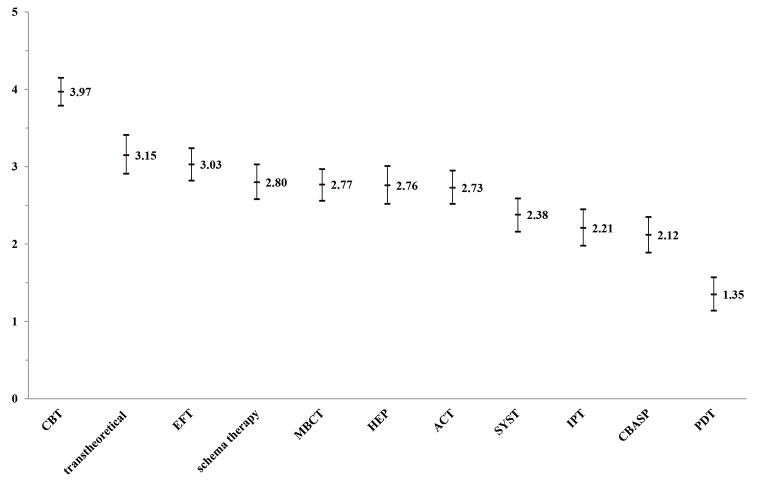
Therapeutic Orientation Within *N* = 102 Postgraduate Trainees at the University of Trier *Note.* 0 = not at all; 5 = very much; CBT = cognitive-behavioral therapy; EFT = emotion focused therapy; HEP = humanistic experiental psychotherapy; MBCT = mindfulness-based cognitive therapy; ACT = acceptance and commitment therapy; SYST = systemic therapy; IPT = interpersonal therapy; CBASP = cognitive behavioral analysis system of psychotherapy; PDT = psychodynamic therapy.

Therefore, rather than adhering to a predetermined manualized treatment for a particular diagnosis from the onset of therapy, our approach prioritizes patients’ real-time progress and continuously adapts treatment based on their specific needs (Lutz, Schwartz, & Delgadillo, 2022). Therefore, treatments integrate various therapeutic concepts such as cognitive-behavioral, interpersonal, emotion-, motivation-, and alliance-focused as well as mindfulness- and strength-based interventions. As a result, therapists receive training in disorder-specific manuals, clinical guidelines, and transtheoretical concepts. When surveying our trainees (*N* = 102) about their therapeutic practice (see Figure 2), 80.5%^1^1The following item, "To what extent would you assess your therapeutic work as transtheoretical?" was answered on a scale from 0 (not at all) to 5 (very much). The percentage provided reflects the combined number of trainees who answered 4 and 5 on the scale. identified themselves as transtheoretical with an emphasis on embracing a variety of orientations, while acknowledging that CBT serves as the foundational concept, which, depending on the country, may need to be adapted to different contexts, health care traditions, or legal systems.

Finally, it is crucial to highlight that while there is a considerable level of flexibility in the theoretical portion of training, it is complemented by monitoring patient outcomes via feedback provided by the TTN. Clinical training includes courses on psychotherapy research and its relation to clinical practice as well as the TTN. It is important to recognize that TTN recommendations are data-based and provide probabilities. They must be evaluated with care in a case-specific manner (Lutz et al., in press). This combination of decisions based on clinical and empirical knowledge enables trainees to cultivate their own therapist identity, while working within a framework that prioritizes patient outcomes and knowledge accumulated from psychotherapy research.

Overall, the development of transtheoretical clinical concepts, as illustrated in this paper, holds potential for the future of psychological therapy. By providing an overarching adaptable framework that accommodates both new and traditional schools of thought, along with findings from clinical research, we hope to enable therapists to move beyond the limitations of rigid schoolism and instead embrace a more comprehensive perspective. Aligned with the development of a measurement-based approach to psychological therapy, this framework might improve the accessibility as well as outcome for a broader and more diverse patient population than previously achievable.
